# Section on Ophthalmology, Otology, Laryngology and Rhinology, October 14, 1901

**Published:** 1901-12

**Authors:** A. G. Bennett

**Affiliations:** Secretary


*Section on Ophthalmology, Otology, Laryngology and Rhinology, October 14, 1901.*

REPORTED BY A. G. BENNETT, M. D., Secretary.

The second regular meeting of the Section of Ophthalmology, Otology, Rhinology and Laryngology was held on Monday evening, October 14, 1901 in the Academy rooms.

Dr. GEO. F. COTT read a paper on the Non-surgical treatment of some of the commonest forms of deafness. (See page 349.)

Dr. CASEY A. WOOD, of Chicago, read a paper on the Eye symptoms of exophthalmic goitre. (Will appear in full in subsequent number of the JOURNAL.)

Dr. ALVIN A. HUBBELL said he objected to the use of proper names applied to diseases, as the custom confused the student. He agreed with Dr. Wood as regards the theory of intoxication, and believed that treatment carried out on these lines would eventually prove the successful method.

Dr. JAMES W. PUTNAM spoke of the influence of fright and emotion. He related a case of railroad accident which was followed by exophthalmic goitre, and a second in which a fireman was notified of his father's sudden death, the shock of which induced the disease. He also instanced cases in which the emotions, such as fear, stage fright, and the like, produced marked effect on the secrétions. His experience is that the exophthalmos is a late symptom.

Dr. MARCEL HARTWIG mentioned the success of Jonnesco's operation of the removal of the cervical sympathetic, but because of Claude Bernard's experiments, which showed shrinkage of the brain, would hesitate to recommend it.

Dr. ELMER STARR, chairman, reported a case in which optic neuritis occurred, followed by mania and death in three weeks. Autopsy showed tumor in occipital region.

Dr. A. G. Bennett reported a case in which the exophthalmos was limited to the left eye and in which the eye was lost from corneal necrosis.

Dr. Wood in reply said he believed in the emotional theory, as he had seen such a case. He believed in the hygienic treatment rather than drug method, and that when the medical treatment failed surgery should be employed. He thought that the Jonnesco operation should be advised.

Dr. Lucien Howe moved a vote of thanks to Dr. Wood, which was carried.

Dr. George F. Cott then reported a case of mastoid disease, in which he had cut down on the lateral sinus, and removed a large thrombus—specimen shown—with a result of perfect recovery.

Meeting adjourned at 10.45 p.m.

